# Inhibition of Fatty Acid Synthesis Aggravates Brain Injury, Reduces Blood-Brain Barrier Integrity and Impairs Neurological Recovery in a Murine Stroke Model

**DOI:** 10.3389/fncel.2021.733973

**Published:** 2021-08-16

**Authors:** Lisa Janssen, Xiaoyu Ai, Xuan Zheng, Wei Wei, Ahmet B. Caglayan, Ertugrul Kilic, Ya-chao Wang, Dirk M. Hermann, Vivek Venkataramani, Mathias Bähr, Thorsten R. Doeppner

**Affiliations:** ^1^Department of Neurology, University Medical Center Göttingen, Göttingen, Germany; ^2^Regenerative and Restorative Medical Research Center, Istanbul Medipol University, Istanbul, Turkey; ^3^The Institute of Translational Medicine, Shenzhen Second People’s Hospital, The First Affiliated Hospital of Shenzhen University, Shenzhen, China; ^4^Department of Neurology, University of Duisburg-Essen, Essen, Germany; ^5^Department of Medicine II, University Hospital Frankfurt, Frankfurt, Germany; ^6^Institute of Pathology, University Medical Center Göttingen, Göttingen, Germany

**Keywords:** blood-brain barrier, cerebral ischemia, fatty acid synthesis, hypoxia, neuroprotection, reduction potential

## Abstract

Inhibition of fatty acid synthesis (FAS) stimulates tumor cell death and reduces angiogenesis. When SH-SY5Y cells or primary neurons are exposed to hypoxia only, inhibition of FAS yields significantly enhanced cell injury. The pathophysiology of stroke, however, is not only restricted to hypoxia but also includes reoxygenation injury. Hence, an oxygen-glucose-deprivation (OGD) model with subsequent reoxygenation in both SH-SY5Y cells and primary neurons as well as a murine stroke model were used herein in order to study the role of FAS inhibition and its underlying mechanisms. SH-SY5Y cells and cortical neurons exposed to 10 h of OGD and 24 h of reoxygenation displayed prominent cell death when treated with the Acetyl-CoA carboxylase inhibitor TOFA or the fatty acid synthase inhibitor cerulenin. Such FAS inhibition reduced the reduction potential of these cells, as indicated by increased NADH_2_^+^/NAD^+^ ratios under both *in vitro* and *in vivo* stroke conditions. As observed in the OGD model, FAS inhibition also resulted in increased cell death in the stroke model. Stroke mice treated with cerulenin did not only display increased brain injury but also showed reduced neurological recovery during the observation period of 4 weeks. Interestingly, cerulenin treatment enhanced endothelial cell leakage, reduced transcellular electrical resistance (TER) of the endothelium and contributed to poststroke blood-brain barrier (BBB) breakdown. The latter was a consequence of the activated NF-κB pathway, stimulating MMP-9 and ABCB1 transporter activity on the luminal side of the endothelium. In conclusion, FAS inhibition aggravated poststroke brain injury as consequence of BBB breakdown and NF-κB-dependent inflammation.

## Introduction

After more than twenty years of intensive research activities, the pathophysiology of cerebral ischemia is still poorly understood (Nakamura and Shichita, 2019). Significant contributions have been made in the past, deciphering fundamental processes of the pathophysiology of cerebral ischemia such as excitotoxicity and inflammation (Dirnagl et al., 1999; Levine, 2004). Nevertheless, these approaches display a mechanistic and oversimplified view of the complex signaling cascade that is activated upon induction of cerebral ischemia. Such limited knowledge is therefore likely the reason why translational stroke research has failed until recently (Nakamura and Shichita, 2019). Additional experimental research beyond analyzing injurious signaling cascades of the ischemic brain and its periphery is of uttermost importance, but both adaptation and preservation mechanisms of the ischemic neuron have long been neglected. Hence, previous work by Brose et al. (2016) sought out to study whether or not fatty acids are critically involved in the adaption process of neurons exposed to *in vitro* hypoxia.

The synthesis of fatty acids plays a pivotal role in cell metabolism. Considering their long-chained structure, they contain vast amounts of electrons that serve as energy storage. Fatty acids represent an essential compound of cell membranes, thus forming the cell surface and stability as well as the cell compartments. Fatty acid synthesis (FAS) is conducted by two enzymes, i.e., Acetyl-CoA carboxylase and fatty acid synthase, with the former acting as the rate-limiting enzyme. After several steps of elongation, reduction and condensation, one palmitate molecule is synthesized from seven molecules of malonyl-CoA involving NADPH as a cofactor (Rassow, 2006; Heinrich et al., 2014).

In recent years, elucidating the role of fatty acids during hypoxia and/or ischemia has been brought to attention by inhibiting FAS. Experimental designs inhibiting FAS under hypoxic conditions are scarce and almost exclusively restricted to research related to oncology. In such studies, FAS is compensatorily activated in neoplastic cells that suffer from local hypoxia due to their extensive proliferation rate (Zhao et al., 2006; Svensson et al., 2016). Hence, the inhibition of FAS by cerulenin or 5-(tetradecyloxy)-2-furoic acid (TOFA) enhances apoptotic cell death in neoplastic cells to up to fifty percent (Matsumae et al., 1963; Omura, 1976; Zhao et al., 2006).

Using an *in vitro* stroke model on neuronal cells with up to 42 h of hypoxia in glucose-rich cell culture medium, Brose et al. (2014, 2016) analyzed the contribution of FAS on the survival of such hypoxic cells. Primary neurons and SH-SY5Y cells exposed to hypoxia displayed increased FAS, where glutamate and glutamine have been incorporated into lipids at significantly high levels (Brose et al., 2014). Inhibition of FAS in hypoxic SH-SY5Y cells, on the contrary, significantly increased the cell death and revealed an elevated ratio of NADH_2_^+^/NAD^+^ (Brose et al., 2016). The authors concluded that fatty acids may indeed serve as hydrogen acceptors upon induction of hypoxia, thus maintaining the cell reduction potential of neurons under these conditions.

Extending the research performed by Brose et al. (2014, 2016), the present work analyzed whether or not a significant role for FAS exists in an *in vitro* hypoxia/reoxygenation model and under *in vivo* stroke conditions. The manuscript concludes with an extensive analysis of possible mechanisms being involved in FAS inhibition under stroke conditions with special emphasis on cell survival, blood-brain barrier (BBB) leakage, inflammation, and neurological recovery.

## Materials and Methods

### Cell Culture of Primary Neurons and SH-SY5Y Cells

Female mice were sacrificed at 16.5 days of pregnancy via CO_2_ inhalation. The abdominal cavity was opened, and the embryos were removed from the uterus. After decapitation, the scalp was removed, and the skull incised in a sagittal manner. The two cortices were divided along the corpus callosum, followed by a dissection of the cerebellum and the olfactory bulb. After removal of the meninges, the cortices were transferred into ice cold PBS. For isolation of the cortex cells, the PBS was removed, and the cortices were incubated in 1 ml of trypsin for 15 min at 37°C. After 14 min, a volume of 50 μl of DNAse was added. Afterward, the mixture was centrifuged for 1 min at 800 rpm, the supernatant removed, 1 ml FCS added, and the tissue was triturated by pipetting gently up and down. After further centrifugation, the cells were resuspended in neurobasal medium (Gibco, Darmstadt, Germany) with additional transferrin (Sigma-Aldrich, St. Louis, MO, United States), penicillin/streptomycin (PS; Gibco, Darmstadt, Germany), L-glutamine (Seromed, Dollnstein, Germany), and B27 supplement (Gibco, Darmstadt, Germany). The cells were counted and seeded at a density of 100,000 cells/cm^2^. Three days after seeding, the cells were used for experiments.

SH-SY5Y cells were passaged every 3–4 days. After removal of the medium, the cells were washed with PBS and incubated for 1 min at 37°C with trypsin. For blockage of the reaction, FCS was added, and the detached cells were transferred into a tube followed by centrifugation for 5 min at 1,200 rpm. The cells were then washed once with PBS and centrifuged again. After resuspension of the cell pellet in neurobasal medium, they were transferred into a new cell culture flask. Given 24 h of resting, the cells were used for experiments.

### Cell Culture of Primary Endothelial Cells and bEnd.3 Cells

Mouse brain endothelial cells (bEnd.3, CRL-2299^TM^, American Type Culture Collection, Manassas, Virginia, United States) were seeded in TC-plates (Sarstedt, Nuembrecht, Germany) and cultured under confluent conditions at a density of 6 × 10^4^ cells/cm^2^. Cells were cultured with 10% fetal bovine serum-containing medium (Dulbecco’s Modified Eagle Medium/Ham’s F-12, Biochrom GmbH, Berlin, Germany).

Primary mouse brain endothelial cells were isolated according to a previously published protocol (Assmann et al., 2017). Briefly, four male C57BL/6 mice were sacrificed and whole brains were removed and stored in HBSS buffer on ice. The brain stem, cerebellum and meninges were removed, thereafter. The cortical tissue homogenate was pelleted by centrifugation at 1,350 × g for 5 min at 4°C, and the pellet was resuspended in 10 ml dextran solution and vortexed extensively (2 min). The mixture was pelleted by centrifugation at 3,900 × g for 10 min at 4°C, and the pellet was resuspended in pre-warmed digestion medium and incubated at 37°C for 1 h with gentle shaking. After digestion, the microvessel fragments were pelleted by centrifugation at 1,350 × g for 5 min at room temperature and washed once in PBS. The resulting microvessel fragments were resuspended in full medium [DMEM/F12 with 20% plasma-derived serum (PDS), L-glutamine, heparin, puromycin, antibiotic/antimycotic, and endothelial cell growth supplement (ECGS)]. The endothelial cells were cultivated on 10 μg/cm^2^ collagen IV-coated flasks, plates or Transwell systems at 37°C.

### Cell Culture of Primary Astrocytes

Primary mouse brain astrocytes were prepared using a previously published protocol (Schildge et al., 2013). Four C57BL/6 mouse pups at postnatal day 0–2 were decapitated and whole brains were treated as described before. The meninges were dissected from the cortex hemispheres by pulling with a forceps. The pooled cortical tissue was digested with 0.25% trypsin at 37°C for 30 min. The cell suspension was then centrifuged at 300 × g for 5 min, and the resulting pellet was suspended in 20 ml of astrocyte full medium (DMEM supplemented with 10% FBS and 1% penicillin/streptomycin) and cultivated on Poly-D-lysine (PDL)-coated T75 flask. The astrocytes were cultured for 1 week (37°C, 5% CO_2_) before a full medium change to remove non-adherent cell debris. The cells were passaged with 0.25% trypsin and resuspended in full medium at a density of 6 × 10^4^ cells/cm^2^.

### Oxygen-Glucose-Deprivation (OGD) Injury

Cells were exposed to OGD when they reached 90% confluence. For OGD, the cells were incubated in BSS0 solution (116 mM NaCl, 5.4 mM KCl, 0.8 mM MgSO_4_, 1 mM NaH_2_PO_4_H_2_O, 26.2 mM NaHCO_3_, 10 mM HEPES, 0.01 mM glycine and 1.8 mM CaCl_2_, pH 7.3) and transferred to a hypoxia incubator chamber containing 0.2% O_2_, 5% CO_2_, and 70% humidity (Toepffer Lab Systems, Göppingen, Germany). For reoxygenation after removing the BSS0 solution, the cells were incubated in their proper cell culture medium for 24 h in the 5% CO_2_ incubator at 37°C. For cell viability assays, neuronal cells were exposed to OGD for either 10 h (primary neurons and SH-SY5Y cells) or for 16 h (bEnd.3). While using the co-culture system (see below), the experimental parameters differed with cells being exposed to OGD for 24 h; primary endothelial cells possessed a more robust tolerance than bEnd.3 cells in our hands.

### Cell Viability

For cell viability measurement, cells were seeded in 24-well plates at a density of 200,000/well using a previously published protocol (Zhang et al., 2021). At the end of the reoxygenation period, 100 μl MTT solution (5 mg/ml dissolved in PBS) were added to each well and incubated for 3.5 h at 37°C. Afterward, the supernatant was removed from each well and replaced by 500 μl DMSO per well. The plates were placed on a plate shaker for 5 min and 100 μl of each well were transferred to a microtiter plate. The analysis was performed by an absorbance microplate reader (TECAN Sunrise) at 565 nm absorption via the software Magellan (TECAN).

### *In vitro* Co-culture BBB Model and Transcellular Electrical Resistance (TER) Measurement

A valid *in vitro* co-culture BBB model was used consisting of primary endothelial cells and astrocytes as described by our group before (Zhang et al., 2021). The endothelial cells were seeded on a microporous membrane in the upper compartment, whereas astrocytes were put in the bottom compartment. As such, both the luminal and the abluminal side of the BBB are represented. When astrocytes were grown to 90% confluence, astrocytes were passaged with 0.25% trypsin and seeded into Poly-D-lysine (PDL)-coated 24-well metal plates of the cellZscopeE instrument (nanoAnalytics, Münster, Germany). These were prepared at least 3 days before the inserts with endothelial cells were placed to the 24-well plate. Endothelial cells grown on Collagen IV-coated inserts (0.4 μm pore diameter, translucent, Greiner Bio-One GmbH, Frickenhausen, Germany) were transferred to the cellZscopeE instrument to establish a co-culture system 2 days after isolation of the endothelial cells. Puromycin was added to the full medium during these 2 days in order to remove non-endothelial cells. After the start of the experiment, the TER values of the barrier were measured automatically under different treatment paradigms by means of impedance measurement (Eigenmann et al., 2013; Czupalla et al., 2014; Kuzmanov et al., 2016; Maherally et al., 2018). No TER measurement was performed during the OGD period itself due to technical limitations.

### BBB Permeability *in vitro* Assays With Evans Blue Albumin (EBA) and Lucifer Yellow (LY)

The flux of different sized molecules like LY and EBA across the endothelial cell membrane of the aforementioned *in vitro* BBB model was analyzed as previously described (Takata et al., 2013; Zhang et al., 2021). Cell culture inserts were transferred to 24-well plates containing an 0.8 ml permeability assay buffer (141 mM NaCl, 2.8 mM CaCl_2_, 1 mM MgSO_4_, 4 mM KCl, 1 mM NaH_2_PO_4_, 10 mM glucose and 10 mM HEPES, pH 7.4) in the bottom (abluminal) compartment. In the inserts (luminal compartment), the culture medium was replaced by 0.2 ml buffer containing 50 μM LY (MW: 457.25 Da) or 4% bovine serum albumin mixed with 0.67 mg/ml EBA (MW: 67,000 Da). Samples (200 μl) were collected from each bottom well at 15, 30, 45, 60, and 120 min to a 96-well plate for the next detection. After removing the samples, each well received fresh permeability assay buffer. The concentrations of LY at different time points were determined with a POLARstar Omega Multimode Plate Reader (BMG LABTECH GmbH, Ortenberg, Germany) using a fluorescein filter pair [Ex(λ) 485 ± 10 nm; Em(λ) 530 ± 10 nm]. The EBA concentration of the abluminal chamber at different time points was measured by determining the absorbance of samples at 630 nm photometrically (Eigenmann et al., 2013; Takata et al., 2013; Watson et al., 2013; Maherally et al., 2018; Yang et al., 2018). The transendothelial permeability coefficient P_cells_ was calculated as given by Dehouck et al. (1992) and Zhang et al. (2021) with slight modifications.

### Quantification of Fatty Acids

For this analysis, 6-well plates with 1 × 10^6^ cells per well were applied using the Free Fatty Acid Quantification Kit (Abcam, Cambridge, United Kingdom). The cells were washed in cold PBS and scratched off. The detached cells were collected in a tube in PBS and centrifuged. Thereafter, the cells were homogenized by adding 200 μl of 1% Triton X-100 in pure chloroform. The mixture was stored on ice for 30 min and centrifuged for 5–10 min. The occurring lower phase was gathered in a new tube and dried at 50°C to eliminate the chloroform. Last traces were removed during 30 min in a vacuum centrifuge. By addition of the fatty acid buffer and intense vortexing, the pellet was resuspended. The standard was prepared as instructed by the manual. For the analysis, 20 μl of each sample were transferred to a test plate and 30 μl of assay buffer added. To each well 2 μl of ACS reagent was added and incubated for 30 min at 37°C. A volume of 50 μl of the reaction mix was added to each well and incubated while being protected from light at 37°C for 30 min. The measurement was administered in a fluorescence microplate reader at an extinction of 544 nm and emission of 590 nm. The quantity of fatty acids was calculated based on the standard curve. Data are given in relation to normoxia controls, which were set as 1.

For *in vivo* measurement, the animals were sacrificed on the seventh day. After preparation of the brain, the brainstem was removed and the hemispheres divided. The ischemic hemispheres (or sham hemispheres) were each washed in cold PBS and homogenized by a bead mill (TissueLyser LT, Qiagen) after addition of 200 μl 1% Triton X-100 in pure chloroform. Further analysis was performed as explained for the cell experiments above. Data are given in relation to hemispheres from sham animals, which were set as 1.

### Analysis of NADH_2_^+^/NAD^+^ Ratio

The NADH_2_^+^/NAD^+^ ratio was analyzed using 6-well plates and the NADH_2_^+^/NAD^+^ Assay Kit (fluorometric; Abcam, Cambridge, United Kingdom). After reoxygenation, the cells were washed in cold PBS, the detached cells collected in a tube and centrifuged for 5 min at 1,500 rpm. The supernatant was discarded, and the cell pellet resuspended in 100 μl lysis buffer. After incubation for 15 min at room temperature, the mixture was centrifuged again at 1,500 rpm for 5 min, and the supernatant was used for the analysis. The standard was prepared according to the manual. On the test plate, 75 μl of reaction mixture were added to every well and incubated for 1.5 h protected from light. The analysis was performed in a fluorescence microplate reader at ex/em = 544/590 nm.

For assessment of the NADH_2_^+^/NAD^+^ ratio *in vivo*, animals were sacrificed 7 days poststroke, and the brains were removed. After dissecting the brain stem, the hemispheres were divided and washed in cold PBS. Next, the hemispheres were homogenized separately in 400 μl lysis buffer. The homogenate was centrifuged for 10 min at 2,500 rpm and the supernatant transferred into a new tube. The procedures thereafter were analog to the cell experiments.

### Experimental Groups

All experiments were approved by local authorities, following the ARRIVE and STAIR guidelines. Male C57BL6/J mice, obtained from Janvier Labs, were included into the experiments at the age of 8–10 weeks. The mice were housed under a circadian rhythm and were supplied with food and water *ad libitum*. Using a power calculation with the value of 0.8 and an effect size between 0.25 and 0.4, mice were randomly allocated to the experimental treatment groups. All experiments were performed in a blinded fashion.

### Induction of Middle Cerebral Artery Occlusion (MCAO)

After injection of buprenorphine (0.1 mg/kg body weight) 30 min prior to the start of the surgery, the animals were anesthetized by 1.5–2% isoflurane. To monitor the cerebral blood flow, a doppler probe (Perimed, Sweden) was attached to the cranium above the territory of the middle cerebral artery (MCA). By an incision at the ventral neck and division of the thymus, the common carotid artery was exposed and ligated at proximal and distal places. Another ligature knot was established at the external carotid artery. A clip was placed on the internal carotid artery, followed by an incision of the common carotid artery. Simultaneously to releasing the clip, a silicon-coated filament (Doccol, Sharon, MA, United States) was inserted into the incision toward the internal carotid artery and further to the middle cerebral artery. The filament was removed after 60 min. Starting at the beginning of reperfusion, daily intraperitoneal injections of cerulenin (50 mg/kg body weight) dissolved in DMSO were given once on each consecutive day for the remainder of the experiment.

### Infarct Volumetry

On the fourth day after induction of MCAO, the animal was sacrificed through deep anesthesia and cervical fracture. By decapitation and opening of the skull, the brain was removed. Using a brain matrix, the brain was cut into slices of 2 mm. For staining of the vital tissue, the slices were incubated in a solution of 2% triphenyltetrazoliumchloride (TTC) in PBS for 10 min. To measure the infarct volume, the slices were scanned and analyzed using the software ImageJ (National Institutes of Health, Bethesda, United States).

### Immunohistochemistry

Immunohistochemical analyses were performed on days 7 and 28 after induction of stroke. At the time points given, the animals were perfused transcardially with 4% paraformaldehyde. The brains were cryopreserved, and coronal sections of 12 μm thickness cut and stored at −20°C until usage. For preparation of staining, the sections were defrosted at room temperature and afterward kept in ice-cold acetone. Thereafter, the slides were washed and heated in a microwave. After several washing steps, the sections were incubated with the primary antibody over night at 4°C. The following primary antibodies were applied: polyclonal rabbit anti-Iba1 (1:500, WAKO 019-19741, Osaka, Japan), polyclonal chicken anti-GFAP (1:1,000, Millipore AB 5541, Nottingham, United Kingdom), and a monoclonal mouse anti-NeuN (1:1,000; Millipore). The day after, the slides were washed, and the following secondary antibodies were applied: goat anti-chicken Alexa Fluor (1:250, 103-547-008, Immuno Jackson, Ely, United Kingdom), goat anti-mouse Alexa Fluor (1:500, Thermo Fisher Scientific), and donkey anti-rabbit Cy3 (1:250, 711-165-152 Immuno Jackson). For nuclear staining, 250 μl of DAPI in TBS at a concentration of 1:10,000 were added to a last washing step and incubated for 10 min at room temperature.

The Terminal deoxynucleotidyl transferase (TdT)-mediated dUTP nick end labeling (TUNEL) staining was performed using the *in situ* cell death detection Kit, TMR red (Sigma Aldrich) on day 7. The tissue was fixated in 4% paraformaldehyde in PBS (pH 7.4) for 20 min and washed afterward. To enhance the permeability, the slides were incubated in citrate buffer in a microwave. After washing, 50 μl of TUNEL reaction mixture were added to each section and incubated for 60 min at 37°C protected from light. All immunohistochemical cell count analysis was performed within the ischemic basal ganglia of the ischemic was done at 0.14 mm anterior, 2.5–3.25 mm ventral and 1.5–2.25 mm lateral from bregma.

### Evans Blue Extravasation

BBB integrity was evaluated by Evans blue extravasation, which was performed as previously described (Doeppner et al., 2011; Radu and Chernoff, 2013). Briefly, 100 μl of 2% Evans Blue dye (Sigma-Aldrich, Darmstadt, Germany) was administered via the femoral vein 2 h before sacrifice. Subsequently, the mice were sacrificed and transcardially perfused with PBS. The ischemic hemispheres were weighed, homogenized in 2 mL of 50% trichloroacetic acid, and centrifuged at 10,000 rpm for 20 min. The extracted Evans Blue dye was further diluted with ethanol, and the absorbance at 620 nm wavelength was measured photometrically. The Evans Blue concentration was based on a standard curve (2.5–500 ng/ml) calculated. Evans Blue extravasation of each group was evaluated, which is given as (μg) Evans Blue per (g) tissue.

### Gelatin Zymography

The gelatin zymography measurement was performed with affinity-support purification with (pro-)MMP-9 serving as standards as previously described (Zhang and Gottschall, 1997; Hu and Beeton, 2010). In short, hemispheres were lysed in a non-reducing lysis puffer containing 50 mM Tris-HCl (pH 7.6), 150 mM NaCl, 5 mM CaCl2, 0.05% BRIJ-35, 0.02% NaN3 and 1% Triton X-100, and afterward centrifuged at 12,000 rpm for 5 min. After collecting the supernatant, the protein concentration was measured (Pierce^TM^ BCA Protein Assay Kit, Thermo Fisher Scientific) and the samples were incubated with 1:10 volume of sepharose 4B (Sigma-Aldrich, Darmstadt, Germany) for 60 min at 4°C. After incubation and centrifugation, the purified pellet was resuspended in lysis buffer containing 10% DMSO. Equal amounts of volume were then incubated with non-reducing sample buffer (Carl Roth, Karlsruhe, Germany) and loaded onto 8% polyacrylamide containing 0.1% gelatin. After electrophoresis, the gel was incubated in renaturing buffer containing 2.5% Triton X-100 under gentle agitation for 30 min. The gel was washed twice and incubated for 48 h at 37°C with developing buffer (Novex^TM^). After incubation, the gel was stained with 0.1% Coomassie Blue for 30 min and then destained in washing solution containing 40% methanol and 10% acetic acid. After destaining, a white band remained behind a dark background. Thereafter, the gels were scanned and densitometrically analyzed.

### Western Blots

Samples were lysed in a buffer containing 50 mM Tris, 1% Triton-X 100, 131 mM sodium chloride, 1 mM sodium diphosphate, 1 mM sodium fluoride, 1 mM EDTA, 1% protease inhibitor, and 1% phosphatase inhibitor with a homogenizator for 10 min and subsequently centrifuged at 4°C with 16,000 rpm for 10 min. The supernatant was collected, and quantification of the protein concentration was photometrically accomplished (Pierce^TM^ BCA Protein Assay Kit, Thermo Fisher Scientific). Reducing sample buffer (Carl Roth, Karlsruhe, Germany) was added, and the samples were heated for 5 min at 95°C. Equal amounts of protein were separated on 8–12% SDS-PAGE and transferred onto nitrocellulose membranes (Bio-Rad, California, United States). Following transfer, the membranes were blocked for 1 h and incubated with the primary antibodies against ABCB1 (Abcam, 0.5 μg/ml), NF-κB p65 (Abcam, 0.5 μg/ml), MMP-9 (Abcam, 0.5 μg/ml), β-actin (0.2 μg/ml), and α-tubulin (0.1 μg/ml) overnight. After washing with tris-buffered saline supplemented with 0.1% Tween 20^®^ detergent (TBS-T) three times, the blots were incubated with horseradish peroxidase coupled secondary anti-mouse-antibody and anti-rabbit-antibody (1:10,000) for 1 h. The membranes were bathed in ECL reagent and developed with the imaging system ChemiDoc^TM^ XRS + (Bio-Rad).

### Measurement of Proteasome Activity

The proteasome activity was performed as previously described (Doeppner et al., 2012). The enzyme activity was measured in left ischemic hemispheres from which homogenates were generated 7 days poststroke. The homogenates were made using a lysis buffer that contained 100 mM Tris-HCl, 145 mM NaCl, 10 mM EDTA, and 0.5% Triton X-100 at pH 7.5. The chymotrypsin-like activity of the proteasome was measured using Suc-LLVY-AMC (50 μM; Sigma-Aldrich). The latter was incubated in a volume of 90 μl of reaction buffer [50 mM Tris, 20 mM KCl, 1 mM magnesium acetate, 2 mM dithiothreitol, 1 mM leupeptin, 1 μg/ml aprotinin (Sigma-Aldrich) and 1 mM PMSF (Merck)]. Substrate cleavage was evaluated at 37°C in a fluorescence microtiter plate reader at λ_exc._ = 355 nm and at λ_em._ = 460 nm. During the straight proportional phase of the enzyme kinetics between 8 and 14 min, the delta of such a reaction was calculated. Values are given as arbitrary fluorescence units per min per mg protein, which was determined by means of the Bradford assay.

### Flow Cytometry Analysis

Flow cytometry analysis from brain samples was performed on day 7 poststroke with slight modifications as previously described (Chu et al., 2014; Doeppner et al., 2014b, 2015). Ischemic brain hemispheres were mechanically homogenized in a buffer of collagenase type XI (125 U/ml), hyaluronidase (60 U/ml) and collagenase (450 U/ml) in Ca^2+^/Mg^2+^ supplemented PBS (Sigma-Aldrich). Thereafter, the samples were incubated with the antibody in question as described before (Doeppner et al., 2015). Absolute cell numbers were measured using CountBright counting beads (Invitrogen Life Sciences).

### Behavioral Tests

The behavioral tests were performed as described previously (Doeppner et al., 2014a). For preconditioning, the animals were trained on two consecutive days prior to initiation of the MCAO. Afterward, the mice were tested on days 7, 14, 21, and 28. For the rota rod test, the animal was placed on a treadmill with an accelerating velocity from 4 to 40 rpm. The time was measured until the animal dropped off with a maximum testing time of 300 s.

To examine strength and coordination, the tight rope test was used. The mouse was brought with the front paws toward the middle of a tight rope of 60 cm length, and the time was measured until arrival on one of the platforms at both ends of the rope. While a healthy mouse would bring its hind paws toward the tight rope and use its tail, neurologically impaired animals are unable to do so or would even fall down. The performance was evaluated by a score ranging from 0 (min) to 20 (max).

During the corner turn test, a mouse was placed 10 times between two boards tapering at an angel of 30°. The aim for the mouse was to walk toward the corner until contact to the wall with its whiskers, turning around and walking back. It was surveyed how often the mouse would turn to one side and analyzed by a formula (number left turns – number right turns/10). Animals with a neurological deficit would lateralize to the non-impaired body side, while healthy animals would show no lateralization.

In the balance beam test, fine motor skills and balance are assessed. Each animal was placed on a beam with reducing width, ending with a platform at the end of the beam. The time was measured until the platform was arrived. Out of two measurements the average was taken. For animals unable to reach the platform, a maximum time of 60 s was defined.

### Statistics

Results are shown as means ± SD. All data were normally distributed as indicated by the Kolmogorov-Smirnov test. Accordingly, parametric tests were applied. Statistical analysis was performed using the Student’s *t*-test to compare two groups. The statistical significance of differences between several groups was assessed by a one-way analysis of variance (ANOVA) followed by the Tukey-Kramer’s test for multiple comparisons. Differences were considered significant when *p*-values were less than 0.05.

## Results

### Inhibition of FAS Increases Cell Death Rate in Neurons and Endothelial Cells After OGD Injury

**FIGURE 1:**
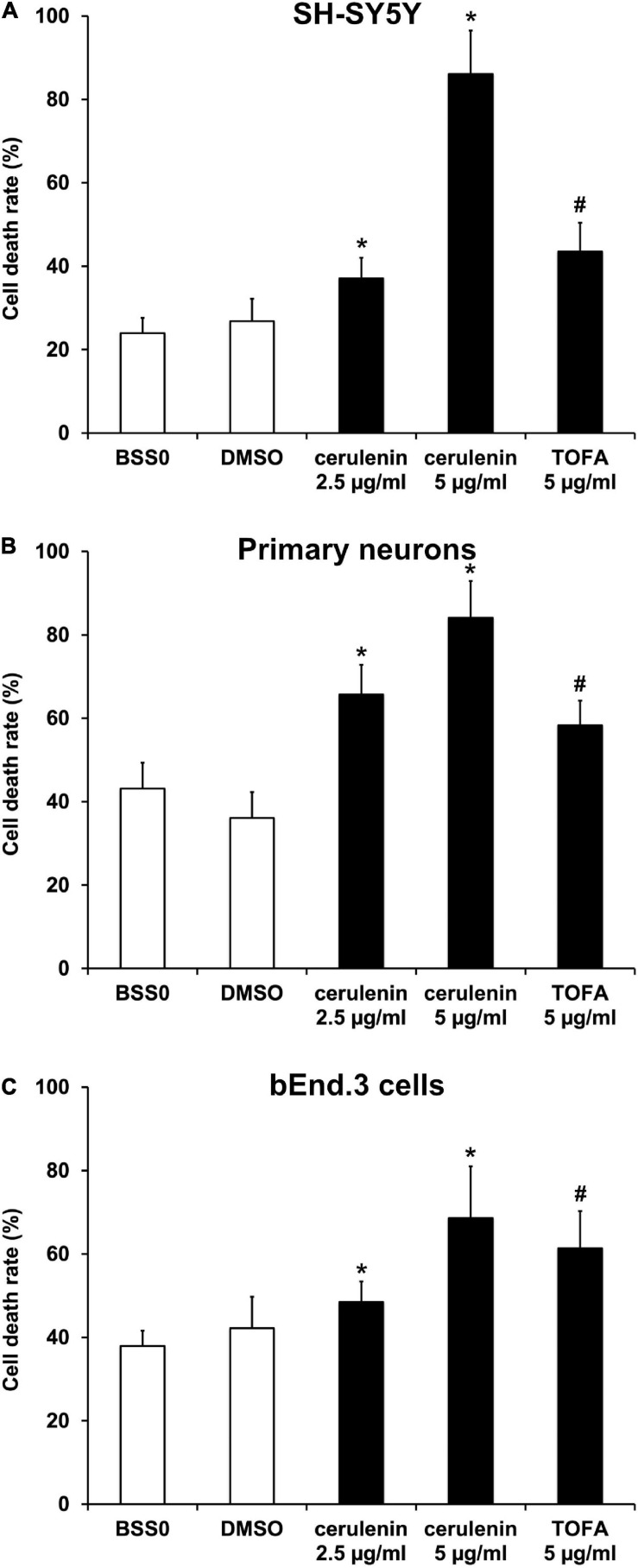
Inhibition of FAS using cerulenin or TOFA aggravates cell death upon induction of oxygen-glucose-deprivation (OGD) *in vitro*. **(A)** Cultivated SH-SY5Y cells, **(B)** primary cortex cells, and **(C)** bEnd.3 cells were exposed to OGD and FAS inhibition by either cerulenin or TOFA (*n* = 4 per condition). The cells were exposed to OGD in BSS0 solution for 10 h **(A,B)** or 16 h **(C)** followed by reoxygenation for 24 h under standard cell culture conditions. BSS0 served as control for TOFA, whereas cerulenin was dissolved in DMSO for which the latter served as control. The cell viability was analyzed using the MTT assay. ^#^Significantly different from BSS0 control, *p* < 0.05. *Significantly different from DMSO control, *p* < 0.05.

Brose et al. (2016) investigated the inhibition of FAS in a model of hypoxia only. The present work therefore applied an OGD model consisting of both hypoxia and reoxygenation. Neuronal cells were exposed to OGD in a glucose-free medium (BSS0) for 10 h followed by 24 h of reoxygenation under standard cell culture conditions. Both SH-SY5Y cells and primary neurons revealed significantly increased levels of fatty acids when exposed to hypoxia (Supplementary Figures 1A,B). Treatment with cerulenin, an inhibitor of the fatty acid synthase, significantly reduced fatty acid concentrations in these cells in a dose dependent manner. Likewise, incubation of cells with TOFA (5 μg/ml), an inhibitor of the Acetyl-CoA carboxylase, also yielded significantly decreased concentrations of fatty acids in SH-SY5Y and primary neurons, albeit to a lesser extent. An analysis of cell death rates in SH-SY5Y cells upon induction of OGD displayed significantly increased cell injury rates. The latter was even further enhanced in the presence of either cerulenin or TOFA (Figure 1A). The results of the tumor cell line were confirmed in primary cortical neurons, i.e., both cerulenin and TOFA increased cell death after OGD when compared to the corresponding controls (Figure 1B). Of note, incubation of these cells with either cerulenin or TOFA did not yield any signs of toxicity under standard, i.e., normoxic cell culture conditions (data not shown).

Regarding clinical aspects, the integrity of the blood-brain barrier (BBB) upon stroke induction is of uttermost importance. Hence, the impact of an inhibition of the FAS in an endothelial cell line was analyzed. When bEnd.3 cells were exposed to OGD, treatment with either cerulenin or TOFA significantly increased cell death in that endothelial cell line (Figure 1C). Again, fatty acid concentrations were increased upon induction of OGD, whereas administration of cerulenin or TOFA significantly reduced fatty acid concentrations. These data thus demonstrate the importance of fatty acids in various cell types under hypoxic conditions. Since both fatty acid concentrations and cell death rates were more affected by high concentrations of cerulenin than by TOFA, cerulenin only was used for the remainder of the study.

### Cerulenin Enhances BBB Leakage and Increases ABCB1 Transporter Abundance in Endothelial Cells Upon OGD Induction

Fatty acids derive from malonyl-CoA, an inhibitor of carnitine palmityl transferase 1, which is located at the outer mitochondrial membrane. That very location is ascribed to the antiapoptotic protein bcl-2, and a mutual activation between the two was previously suggested (Paumen et al., 1997). As a matter of fact, ectopic delivery or overexpression of bcl-2 induces neuroprotection against cerebral ischemia (Kilic et al., 2002; Zhao et al., 2003; Doeppner et al., 2010; Zhang et al., 2015). Inhibition of FAS, on the contrary, leads to reduced endogenous bcl-2 levels, as demonstrated in bladder cancer cells (Jiang et al., 2012). However, the present study did not observe any regulation of bcl-2 in SH-SY5Y or primary cells exposed to OGD treated with either cerulenin or TOFA (data not shown), suggesting that increased cell death rates observed therein has to be mediated by other mechanisms.

Such other mechanisms involve the disruption of the BBB, which allows intravascular molecules and immune cells to penetrate into the extracellular compartment and into the brain parenchyma, reflecting a hallmark in cerebral ischemia (Dirnagl et al., 1999). In this context, various compounds are extruded through the barrier via ATP-binding cassette (ABC) transporters such as ABCB1. The latter is also highly regulated under stroke conditions, contributing to secondary cell injury as shown in preclinical stroke models (Spudich et al., 2006; Zhang et al., 2021). To analyze the impact of inhibition of FAS on the BBB, an *in vitro* co-culture model using endothelial cells and astrocytes was established.

**FIGURE 2:**
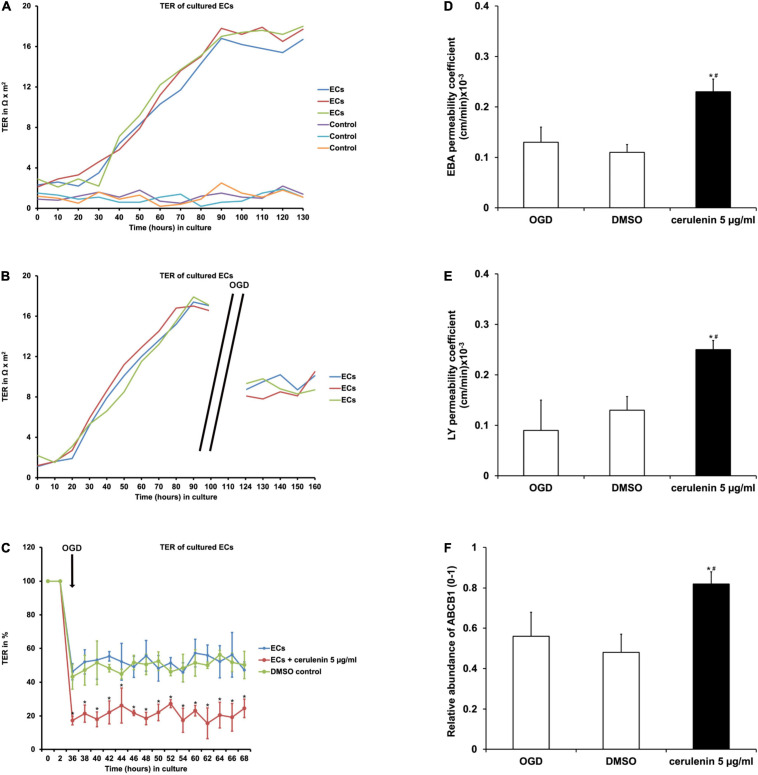
Inhibition of FAS diminishes blood-brain barrier (BBB) integrity and increases the abundance of the ABCB1 transporter in primary endothelial cells. **(A)** Representative graphs depicting the extent of transcellular electrical resistance (TER) in primary endothelial cells (ECs) under standard cell culture conditions. Empty wells, i.e., wells containing no cells at all are referred to as “control,” indicating background noise of the measurement. **(B)** Representative graphs for TER measurement in ECs exposed to oxygen-glucose-deprivation (OGD) injury followed by reoxygenation at standard cell culture conditions. Note that TER measurement was not possible during the OGD period itself due to technical limitations. **(C)** Measurement of TER in primary ECs exposed to OGD (*n* = 4). Some cells were treated with cerulenin, whereas DMSO served as control. Pre-OGD values were set as 100%. **(D,E)** Using a BBB co-culture model existing of both astrocytes and primary endothelial cells (*n* = 4 per condition), permeability coefficients for both Evans blue albumin (EBA; large molecule) and luciferase yellow (LY; small molecule) after OGD exposure were performed (*n* = 4 per condition). Administration of cerulenin significantly increases permeability coefficients in endothelial cells under hypoxic conditions when compared to DMSO controls. In line with this, protein abundance of ABCB1 is significantly increased when fatty acid synthase is blocked by cerulenin in endothelial cells **(F)**. *Significantly different from DMSO control, *p* < 0.05. ^#^Significantly different from OGD samples, *p* < 0.05.

When primary endothelial cells were seeded into 24-wells and kept under standard cell culture conditions, the transcellular electrical resistance (TER) significantly increased over time until it reached a plateau phase, indicating stable formation of the barrier (Figure 2A). Exposure to OGD, however, diminished TER values significantly in these endothelial cells as indicated by the cellZscopeE apparatus (Figure 2B). Administration of cerulenin (5 μg/ml) decreased TER levels upon OGD induction even further (Figure 2C). Mimicking BBB-like features, permeability coefficients were measured in the co-culture system consisting of primary astrocytes and primary endothelial cells. As a matter of fact, both EBA (large molecule) and LY (small molecule) permeability coefficients were significantly increased when cells were treated with cerulenin under hypoxic conditions, indicating enhanced leakage of the “BBB” (Figures 2D,E). Likewise, protein abundance of ABCB1 was significantly increased in the cerulenin group (Figure 2F). This data together with increased cell death rates of endothelial cells exposed to OGD (Figure 1C) is in favor of inhibition of FAS further enhancing leakage of the BBB under *in vitro* conditions.

### Inhibition of FAS Aggravates Brain Injury After Cerebral Ischemia *in vivo*

**FIGURE 3:**
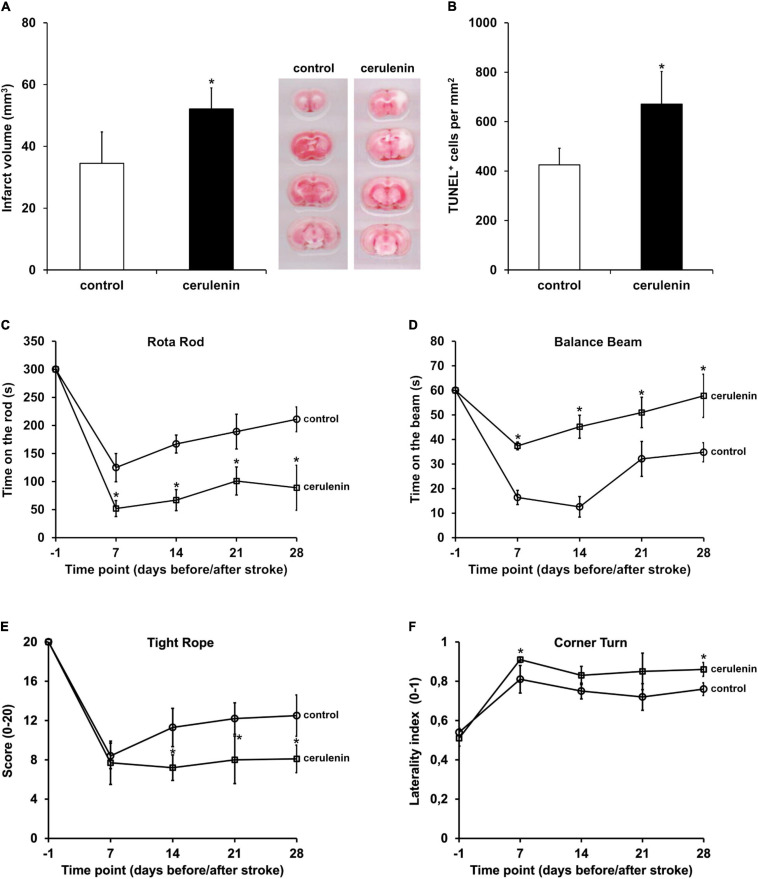
Inhibition of FAS aggravates poststroke brain injury and motor coordination impairment. Mice were either treated with DMSO (control) or with cerulenin (50 mg/kg body weight). Brain injury was assessed using TTC staining (*n* = 6 per condition) on day 4 **(A)** or TUNEL staining (*n* = 8 per condition) on day 7 **(B)**. Behavioral tests (*n* = 21 per condition) were performed during an observation period of 4 weeks at the time points given (circles: controls, squares: cerulenin). The tests included the rota rod **(C)**, balance beam **(D)**, tight rope **(E)**, and corner turn test **(F)**. All animals were trained before induction of stroke in order to ensure proper test performance. *Significantly different from controls, *p* < 0.05.

Following the aforementioned *in vitro* results, the role of FAS was further analyzed in a rodent stroke model. When male C57BL6 mice were exposed to MCAO for 1 h followed by treatment with daily intraperitoneal cerulenin injections (50 mg/kg body weight), mice displayed increased infarct volumes on day 4 poststroke (Figure 3A). The latter was confirmed using TUNEL staining analysis 7 days poststroke (Figure 3B). Long-term analysis revealed that treatment with cerulenin yielded significantly worsened behavioral outcome during the observation period of 4 weeks (Figures 3C–F). The results obtained in the behavioral tests were backed up by an assessment of neuronal densities 4 weeks poststroke (Supplementary Figure 2). Inhibition of FAS significantly reduced neuronal densities in these stroke mice when compared to DMSO controls or sham animals.

### Reduction Potential Is Reduced by Inhibition of FAS

**FIGURE 4:**
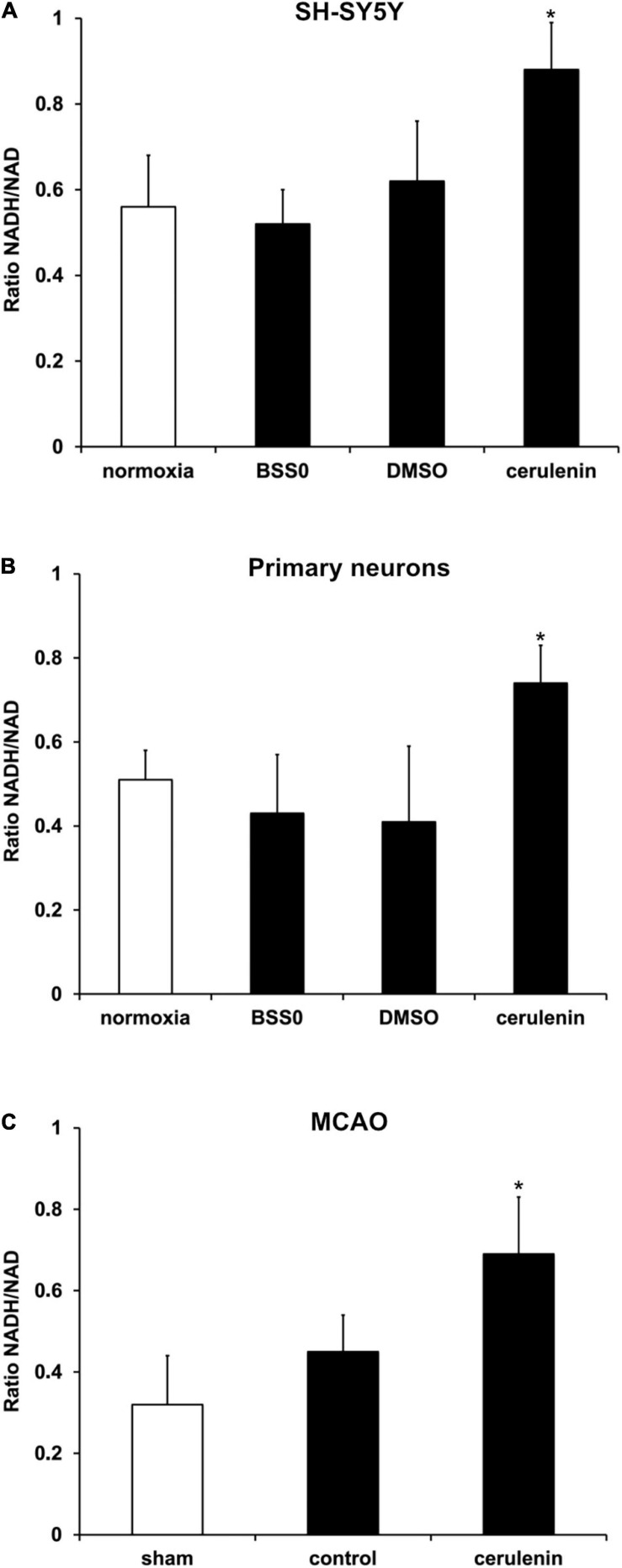
Reduction potential is reduced by inhibition of FAS in SH-SY5Y cells, primary neurons and stroke mice. **(A)** Cultivated SH-SY5Y cells and **(B)** primary cortex cells were exposed to oxygen-glucose-deprivation (OGD) in BSS0 solution for 10 h followed by reoxygenation for 24 h under standard cell culture conditions (*n* = 4 per condition). The cells were either treated with cerulenin or DMSO as control. Some cells were continuously kept under standard cell culture conditions (“normoxia”) as a reference. The NADH/NAD ratio was measured for each condition as given in “Materials and Method” section. **(C)** Measurement of the NADH_2_^+^/NAD^+^ ratio on day 7 after induction of middle cerebral artery occlusion (MCAO) and daily injection of cerulenin (50 mg/kg body weight) in mice (*n* = 6). The control group received injections of DMSO only, whereas sham animals were not exposed to MCAO and did not receive any kind of treatment paradigm. *Significantly different from controls, *p* < 0.05.

Further analyses aimed to answer the question whether or not fatty acid inhibition affects the reduction potential upon induction of OGD *in vitro* or after MCAO *in vivo*. Previous work from Brose and colleagues (Brose et al., 2016) have already shown reduced reduction potentials in neuronal cells under hypoxic conditions. The latter, however, excluded a reoxygenation phase. Herein, exposure of neurons (SH-SY5Y cells and primary neurons) to OGD followed by 24 h of reoxygenation under standard cell culture conditions yielded an increased ratio of NADH_2_^+^/NAD^+^ (Figures 4A,B), confirming the results obtained by Brose et al. (2014, 2016) under these special conditions. Since the reduction potential has not been analyzed under *in vivo* stroke conditions by Brose and colleagues, NADH_2_^+^/NAD^+^ ratios were also studied in stroke animals 7 days after induction of MCAO. Indeed, application of cerulenin also reduced the reduction potential in these animals when compared to DMSO controls (Figure 4C).

### Inhibition of FAS Enhances Poststroke BBB Leakage and Stimulates the NF-κB/Proteasome Pathway *in vivo*

**FIGURE 5:**
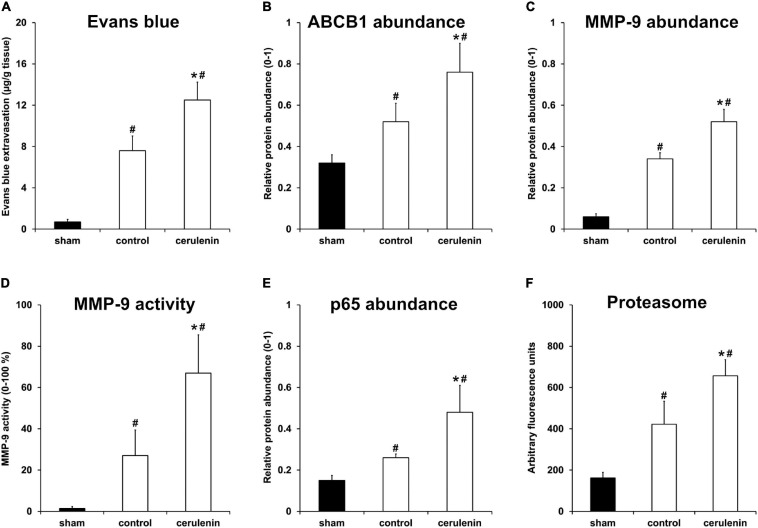
Inhibition of FAS enhances poststroke BBB leakage and stimulates the NF-κB/proteasome pathway *in vivo*. All mice (*n* = 9 per condition) were exposed to middle cerebral artery occlusion (MCAO) followed by cerulenin treatment or DMSO application (controls). Sham mice underwent the same surgical procedure but without MCAO. End of the experiments was 24 h after induction of MCAO. Blood-brain barrier (BBB) leakage was measured via Evans blue extravasation **(A)**, whereas ABCB1 **(B)** and MMP-9 **(C)** abundance was analyzed by means of Western blotting. MMP-9 activity itself was studied using a gelatin zymography assay **(D)**. The NF-κB protein abundance was measured using a Western blot against p65 within the ischemic hemispheres **(E)**. Proteasomal activity was measured using a fluorimetric assay, as described in section “Materials and Methods” **(F)**. ^∗^Significantly different from controls, *p* < 0.05. ^#^Significantly different from sham mice, *p* < 0.05.

Application of cerulenin enhances BBB leakage and increases the abundance of the ABCB1 transporter under *in vitro* OGD conditions (Figure 2). Interestingly, exposure of mice to MCAO followed by treatment with cerulenin also yielded increased extravasation of Evans blue and enhanced the abundance of ABCB1 in these mice on day 1 poststroke (Figures 5A,B and Supplementary Figure 3). The degradation of the BBB upon stroke induction is, however, not only a consequence of the ABCB1 transporter but is also critically mediated by the activity of matrix metalloproteases (MMP) that play in concert with a plethora of other signaling cascades (Lakhan et al., 2013). Indeed, stroke enhanced both protein abundance and activity of MMP-9 at 24 h poststroke when compared to sham mice, which was even further enhanced when mice were treated with cerulenin (Figures 5C,D and Supplementary Figure 3).

Accumulating evidence suggests that ABCB1 transporters as well as MMP-9 activity are regulated by the nuclear factor-kappa B (NF-κB) signaling pathway under non-stroke conditions (Nakanishi and Toi, 2005; Katayama et al., 2014; Chiu et al., 2015; Andersson et al., 2018). Whereas the role of NF-κB and the proteasome under stroke conditions has already been shown (Ridder and Schwaninger, 2009; Harari and Liao, 2010; Doeppner et al., 2013; Lv et al., 2019), the mutual interaction between MMP-9 and ABCB1 on the one hand and NF-κB on the other hand has just recently been described by us in a murine stroke model (Zhang et al., 2021). Whether or not the inhibition of the fatty acid synthase by means of cerulenin under such conditions also interferes with the aforementioned signaling pathways is not yet known. As a matter of fact, stroke itself increased both NF-κB p65 abundance and proteasome activity 24 h after stroke induction (Figures 5E,F and Supplementary Figure 3), as was expected. Treatment of stroke mice with cerulenin, however, further increased NF-κB p65 abundance and proteasome activity when compared to control mice.

### FAS Inhibition Stimulates Poststroke Inflammatory Responses *in vivo*

**FIGURE 6:**
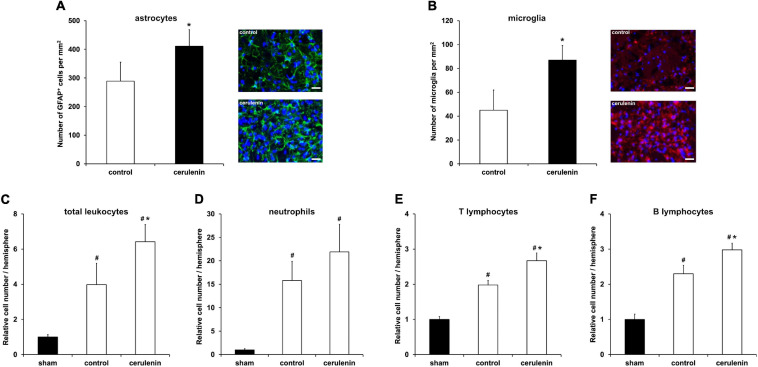
FAS inhibition stimulates poststroke inflammatory responses *in vivo*. Mice were exposed to middle cerebral artery occlusion (MCAO) and allowed to survive for 7 days after induction of MCAO (*n* = 11 per condition). The animals received either cerulenin or DMSO as control, as explained in detail in “Materials and Methods” section. Immunohistochemical analyses for astroglia and microglia was performed using either GFAP (**A**, green) or Iba1 staining (**B**, red). DAPI counterstaining (blue) was performed in parallel. Scale bars are 50 μm. Using flow cytometry, the numbers of total leukocytes **(C)**, neutrophils **(D)**, T lymphocytes **(E)** and B lymphocytes **(F)** were measured on day 7 in ischemic hemispheres. *Significantly different from controls, *p* < 0.05. ^#^Significantly different from sham mice, *p* < 0.05.

Inflammation critically contributes to secondary stroke injury during the subacute phase of the disease as mentioned before. Besides, the NF-κB/proteasome signaling pathway, which is significantly stimulated upon treatment with cerulenin (Figure 5), regulates proinflammatory cell cascades on its own (Phillips et al., 2000; Zhang et al., 2010, 2016; Doeppner et al., 2015). Indeed, inhibition of FAS not only resulted in increased glial scar formation (Figure 6A) and microglial response (Figure 6B), but also in a significantly enhanced inflammatory reaction within the ischemic hemisphere 7 days after stroke induction. Flow cytometry analyses on that day revealed increased stroke-induced numbers of total leukocytes, neutrophils as well as of B and T lymphocytes (Figures 6C–F). Of note, cerulenin treatment further increased cell numbers of all of these cell populations.

## Discussion

The present work aimed to illuminate whether or not the inhibition of FAS represents a biologically relevant pathway under preclinical *in vitro* and *in vivo* stroke conditions. Although recent findings demonstrated that the inhibition of FAS yields an increased cell death rate and a decreased reduction potential, the work published by Brose et al. (2016) used an *in vitro* model of hypoxia only. Herein, the role of FAS was therefore studied in a hypoxia/reoxygenation model *in vitro* as well as under *in vivo* stroke conditions, emphasizing the role of the BBB and poststroke inflammatory tissue response.

Exposure of SH-SY5Y neuroblastoma cells and primary neurons to *in vitro* hypoxia results in a prominent cell death rate. Irreversible inhibition of fatty acid synthase by covalent binding to the β-ketoacyl synthase domain (Zhao et al., 2006; Furuta et al., 2008; Murata et al., 2010) using cerulenin or inhibition of Acetyl-CoA carboxylase by means of TOFA increases cell death even further. Hence, inhibition of FAS confirms previously published data by Brose et al. (2014, 2016) in our *in vitro* stroke model. The present work, for the first time, demonstrates a biologically relevant role of FAS under *in vivo* stroke conditions. Cerulenin treatment not only resulted in increased histological brain injury upon induction of stroke, but also yielded decreased neurological recovery in these animals during the observation period of 4 weeks.

Nicotinamide adenine dinucleotide (NAD) serves as a cofactor in more than 250 redox reactions, among which are reaction pathways of the glycolysis and the citrate cycle (Heinrich et al., 2014). Under physiological conditions, NAD^+^ is reduced to NADH_2_^+^ followed by its reoxidation while passing the respiratory chain to build up a proton gradient. During oxidative phosphorylation, oxygen is transferred to two protons and as such, one water molecule is generated as a side product. Accordingly, this mechanism and thus the ATP synthesis by oxidative phosphorylation come to a halt under hypoxia. Consequently, NADH_2_^+^ cannot be reoxidated via this pathway. This condition is reflected by the increased ratio of NADH_2_^+^/NAD^+^ under both *in vitro* and *in vivo* stroke conditions as observed in the present work, emphasizing that hypoxia and simultaneous inhibition of FAS negatively affect the cell redox potential. Indeed, application of NAD in a model of transient focal cerebral ischemia has been found to improve neurological outcome (Ying et al., 2007), leading to the assumption that preservation of the redox potential represents a valid goal. These findings are in line with the results by Brose et al. (2016), emphasizing that the inhibition of FAS further limits the potential to reoxidize NADH_2_^+^.

Inhibition of FAS is not only limited to regulating NADH_2_^+^/NAD^+^ ratios but also affects other signaling pathways as well. Fatty acids derive from malonyl-CoA, an inhibitor of carnitine palmityl transferase 1, which itself represents the rate-limiting enzyme of beta oxidation. The close proximity of carnitine palmityl transferase 1 at the outer mitochondrial membrane with the antiapoptotic protein bcl-2 suggest a mutual interaction between the two proteins (Paumen et al., 1997). Bcl-2 has been widely studied in various models of cerebral ischemia where it exerts neuroprotection (Kilic et al., 2002; Zhao et al., 2003; Doeppner et al., 2010; Zhang et al., 2015), and bcl-2 deficient mice display prominent infarct volumes and an aggravated neurological deficit compared to wild type mice (Akhtar et al., 2004). Interestingly, the overexpression of antiapoptotic bcl-2 in glioblastoma cells partly compensates the proapoptotic effect of cerulenin (Zhao et al., 2006). At the same time, data suggests that hypoxia is associated with an increased expression of bcl-2. Inhibition of FAS leads, however, to reduced endogenous bcl-2 levels, as demonstrated in bladder cancer cells (Jiang et al., 2012). Herein, bcl-2 levels were not affected by FAS inhibition, neither in SH-SY5Y cells nor in primary neurons exposed to OGD (own unpublished observation).

Inhibition of FAS is unlikely to affect a single pathway only, if at all, as is the case for bcl-2. Since stroke-induced loss of BBB integrity during the acute phase is of uttermost importance for the course of the disease, studying BBB leakage under both *in vitro* and *in vivo* stroke conditions was analyzed in the presence of FAS inhibition. Whereas some data suggests a role of FAS in the context of endothelial cell proliferation and angiogenesis specifically under neoplastic conditions (Browne et al., 2006; Zaytseva et al., 2014; Bastos et al., 2017; Singh et al., 2017; Bruning et al., 2018), such aspects have not been addressed in endothelial cells exposed to an *in vitro* or *in vivo* stroke model. Not only does inhibition of FAS result in increased cell death rates of endothelial cells under hypoxic but not so under normoxic conditions, which is in line with cytotoxicity studies in tumors, but also result in increased interendothelial cell leakage. The latter is associated with decreased levels of TER in cultured endothelial cells treated with cerulenin under hypoxic conditions, underlining the aggravation of hypoxia-induced BBB breakdown upon inhibition of FAS. On the molecular level, posthypoxic/postischemic BBB breakdown is linked with an upregulation of ABCB1 transporters and MMP-9 activity (Fujimura et al., 1999; Spudich et al., 2006; Park et al., 2009; ElAli and Hermann, 2012; Suofu et al., 2012; Cen et al., 2013; Ji et al., 2013; Qosa et al., 2015; Song et al., 2015; Rempe et al., 2016; Turner and Sharp, 2016; Yang and Candelario-Jalil, 2017; Zhang et al., 2021), and knockdown of ABCB1 or pharmacological inhibition of the transporter reduces poststroke brain injury and ameliorates functional outcome (Spudich et al., 2006; Murozono et al., 2009).

Recent work from our group has established a causal link between increased activity of both ABCB1 and MMP-9 on the one hand and the NF-κB pathway on the other hand under stroke conditions (Zhang et al., 2021). Using a murine stroke model, our group was able to show that stimulation of the proinflammatory NF-κB pathway results in both enhanced MMP-9 activity and increased ABCB1 transporter activity, all of which leading to BBB breakdown, cell death and impaired neurological recovery. The present work was able to show that inhibition of FAS also affects the NF-κB pathway where p65 protein abundance as well as proteasomal activity are significantly increased due to cerulenin treatment. Increased levels of NF-κB-p65 together with enhanced activity of the proteasome, in turn, not only mediate the aforementioned loss of BBB integrity but also activate a plethora of proinflammatory signaling cascades (Phillips et al., 2000; Zhang et al., 2006, 2010; Doeppner et al., 2015). Hence, cerulenin treatment significantly increases microglial activity and enhances overall leukocyte numbers as well as defined subpopulations of leukocytes within the ischemic hemisphere. Such an increased proinflammatory status within the brain due to FAS inhibition further supports secondary brain injury upon stroke induction, culminating in reduced neurological recovery of stroke animals.

The present data for the first time demonstrates the important role of FAS under both *in vitro* and *in vivo* stroke conditions, depicting the fatal consequences of inhibition of FAS with regard to BBB integrity, brain injury, and neurological recovery. Nevertheless, cerulenin application and subsequent FAS inhibition will not only affect the aforementioned signaling cascades but also downstream signaling such as lipid peroxidation. Inhibition of the latter is known to be neuroprotective against stroke, decreasing oxidative stress and further reducing BBB leakage (Yigitkanli et al., 2013; Karatas et al., 2018). In this context, cerulenin does not only irreversibly inhibit fatty acid synthase but also inhibits HMG-CoA synthetase and thus steroid biosynthesis (Nomura et al., 1972), albeit some data indicates that this effect might depend on the species studied (Greenspan and Mackow, 1977). Since both FAS inhibitors used in the present study, i.e., TOFA and cerulenin, pronounced cell injury, it stands to reason that the effects observed herein are attributed to FAS inhibition itself, rather than to side effects of either inhibitor. Yet, additional studies are in order, analyzing the dynamic flux of FAS and bioavailability of fatty acids, both in the central nervous system but also in peripheral organs. Such studies were, however, beyond the scope of the present work.

## Data Availability Statement

The raw data supporting the conclusions of this article will be made available by the authors, without undue reservation.

## Ethics Statement

The animal study was reviewed and approved by the Medipol University Istanbul LAVES.

## Author Contributions

LJ and TD designed and conceptualized the study. XA, XZ, WW, AC, Y-cW, and VV performed experiments, data analysis, and interpretation. EK, TD, and MB provided financial support and infrastructure. LJ, TD, DH, MB, and EK wrote the manuscript. All authors contributed to the article and approved the submitted version.

## Conflict of Interest

The authors declare that the research was conducted in the absence of any commercial or financial relationships that could be construed as a potential conflict of interest.

## Publisher’s Note

All claims expressed in this article are solely those of the authors and do not necessarily represent those of their affiliated organizations, or those of the publisher, the editors and the reviewers. Any product that may be evaluated in this article, or claim that may be made by its manufacturer, is not guaranteed or endorsed by the publisher.
