# Supplementary material for: Inhibition of Fatty Acid Synthesis Aggravates Brain Injury, Reduces Blood-Brain Barrier Integrity and Impairs Neurological Recovery in a Murine Stroke Model
Source: Front Cell Neurosci. 2021 Aug 16;15:733973. doi: 10.3389/fncel.2021.733973 (PMC8415573; doi:10.3389/fncel.2021.733973)
Supplement: Supplementary file 1 [file Data_Sheet_1.PDF]

SUPPLEMENTARY MATERIAL

Inhibition of fatty acid synthesis aggravates brain injury, reduces blood-brain barrier integrity and impairs neurological recovery in a murine stroke model

Lisa Janssen[1], Xiaoyu Ai[1], Xuan Zheng[1], Wei Wei[1], Ahmet B Caglayan[2], Ertugrul Kilic[2], Ya-chao Wang[3], Dirk M. Hermann[4], Vivek Venkataramani[5,6], Mathias Bähr[1], and Thorsten R. Doeppner[1,2]

[1]University Medical Center Göttingen, Department of Neurology, Göttingen, Germany

[2]Istanbul Medipol University, Regenerative and Restorative Medical Research Center, Istanbul, Turkey

[3]The Institute of Translational Medicine, The First Affiliated Hospital of Shenzhen University, Shenzhen Second People's Hospital, Shenzhen, China

[4]University of Duisburg-Essen, Department of Neurology, Essen, Germany

[5]University Hospital Frankfurt, Department of Medicine II, Frankfurt, Germany

[6]University Medical Center Göttingen, Institute of Pathology, Göttingen, Germany

Correspondence to: Thorsten R. Doeppner, M.D.-M.Sc.

Department of Neurology

University Medical Center Göttingen

Robert-Koch-Str. 40

37075 Göttingen, Germany

Phone: +49-551-3966660, fax: +49-551-399348

email: thorsten.doeppner@med.uni-goettingen.de

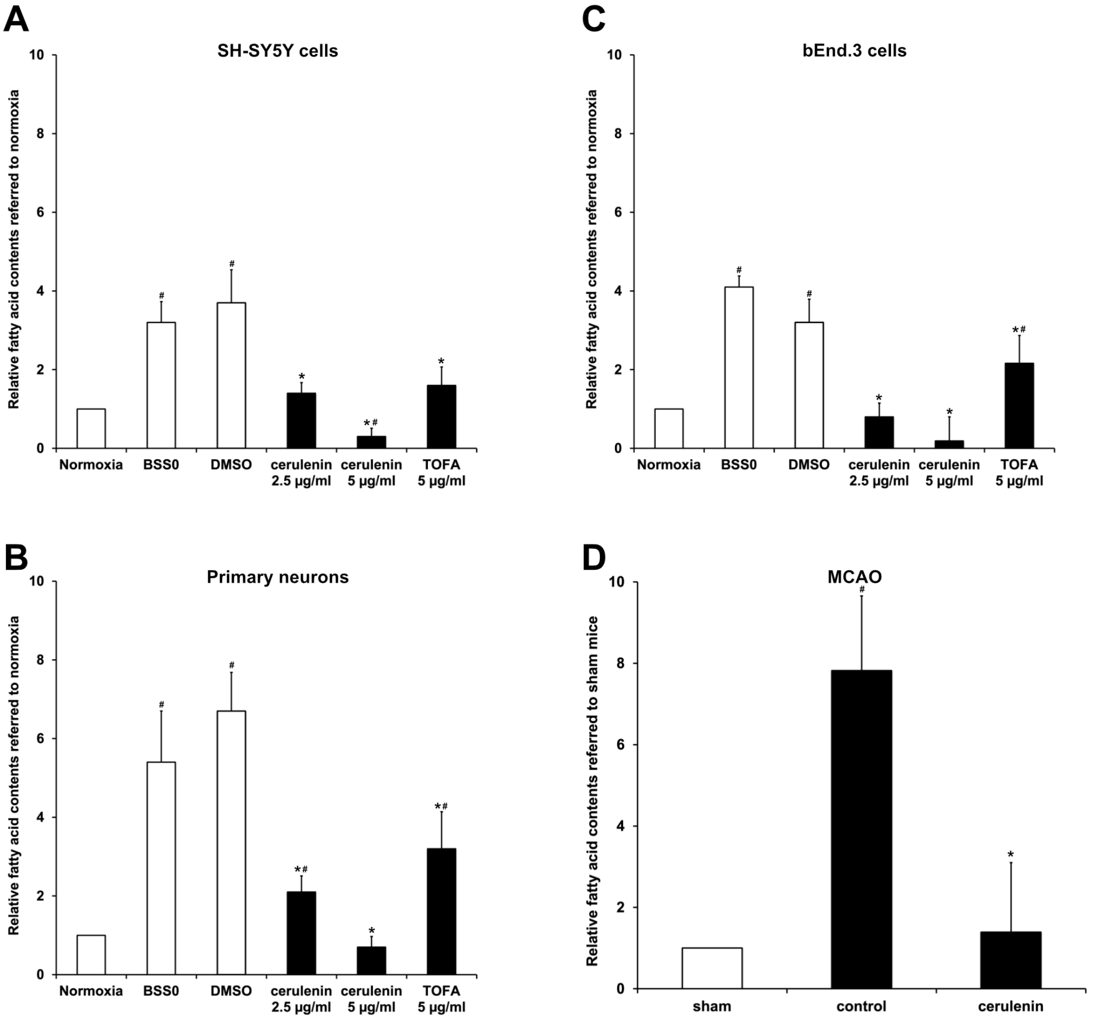

Supplementary Fig. S1. Induction of hypoxia or cerebral ischemia increases fatty acid concentrations. Cells were exposed to hypoxia in BSS0 solution for either 10 h (SH-SY5Y cells **(A)** and primary neurons **(B)**) or for 16 h (bEnd.3 endothelial cells **(C)**) followed by 24 h of reoxygenation under standard cell culture conditions (n=4 per condition). DMSO serves as control for cells treated with cerulenin. Contents of fatty acids is given as relative numbers referring to cells kept under standard (non-hypoxic) cell culture conditions. **(D)** Relative fatty acid concentration referred to sham mice on day 7 after stroke induction (n=6 per condition). Controls received i.p. injections of DMSO as given in the materials and method section. $^{\#}$Significantly different from non-hypoxic ("normoxia") cells or sham mice, p<0.05. *Significantly different from corresponding controls, p<0.05.

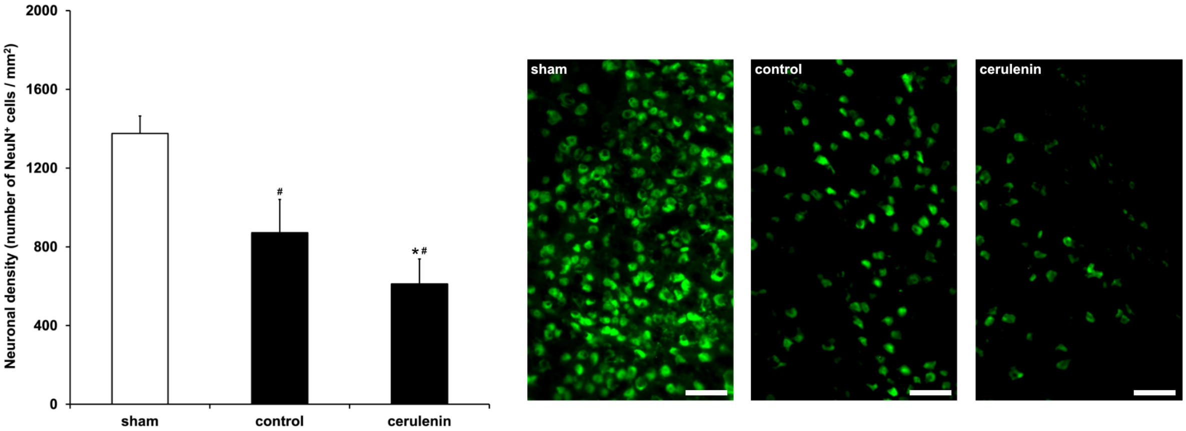

Supplementary Fig. S2. Inhibition of fatty acid synthesis aggravates long-term brain injury. Neuronal densities were analyzed on day 28 poststroke using NeuN staining within the ischemic striatum (n=12 per condition), as given in the materials and method section. DMSO serves as control for mice treated with cerulenin, whereas sham mice were not exposed to cerebral ischemia. Representative photos of such NeuN staining are shown for each condition with scale bars of 50 µm each. $^{\#}$Significantly different from sham mice, p<0.05. *Significantly different from controls, p<0.05.

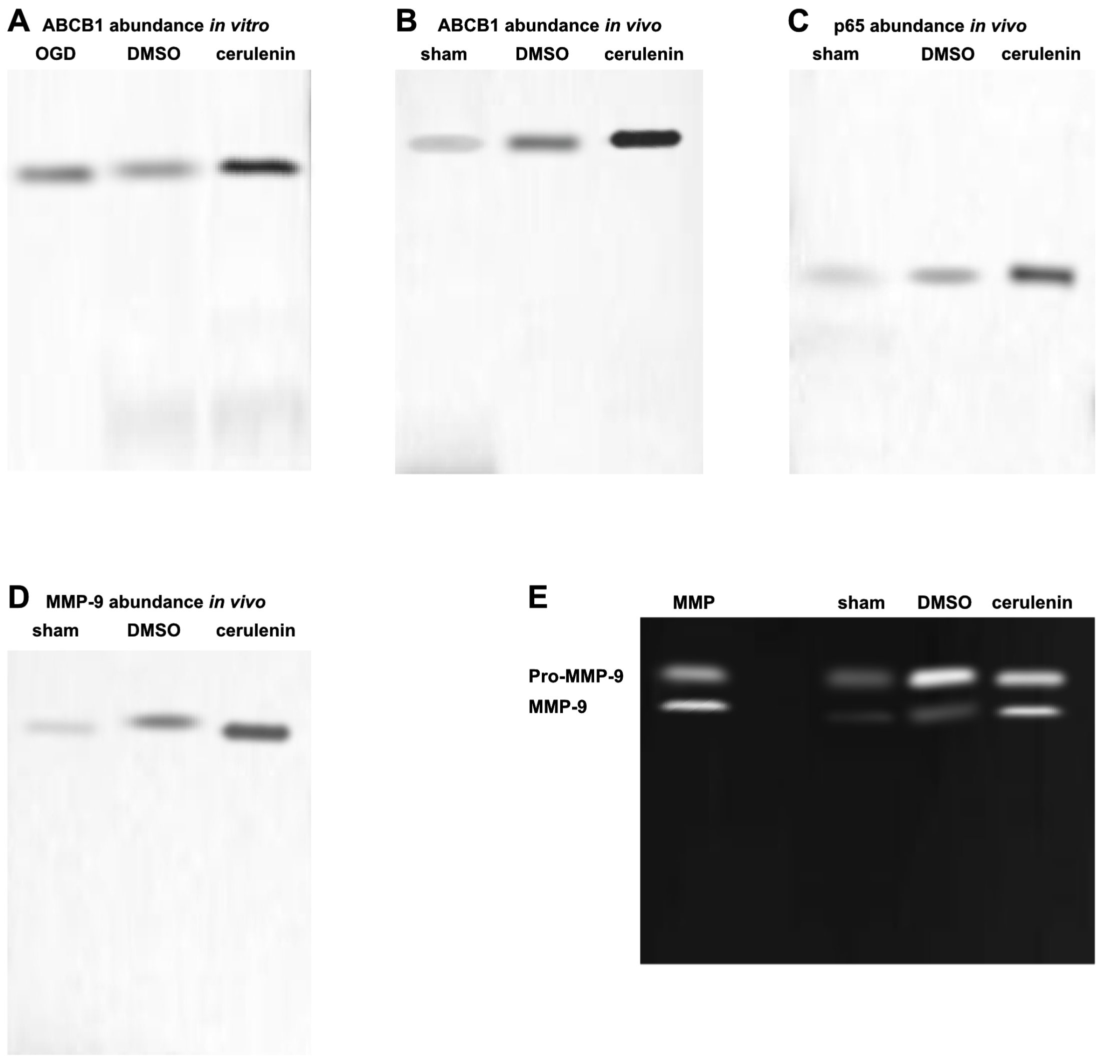

Supplementary Fig. S3. Representative Western blots and gel zymography. Western blot against ABCB1 was done in endothelial cells after oxygen-glucose-deprivation (OGD) injury **(A)** and after middle cerebral artery occlusion (MCAO) in mice **(B)**. The abundance of NF-κB-p65 **(C)** and of matrix metalloprotease (MMP) 9 **(D)** was measured in sham mice and in MCAO mice. MCAO mice were treated with either DMSO (control) or cerulenin. **(E)** Representative gel zymography of MMP-9 in sham and MCAO mice.
